# Supplementary material for: The DNA adenine methylase of Salmonella Enteritidis promotes their intracellular replication by inhibiting arachidonic acid metabolism pathway in macrophages
Source: Front Microbiol. 2023 Mar 2;14:1080851. doi: 10.3389/fmicb.2023.1080851 (PMC10018194; doi:10.3389/fmicb.2023.1080851)
Supplement: Supplementary file 2 [file Data_Sheet_2.zip › S1 Appendix. Non-targeted metabolomics raw data/4.MetDiffAnalysis/C50336_Ddam.vs.control/C50336_Ddam.vs.control_neg_zscore.pdf]

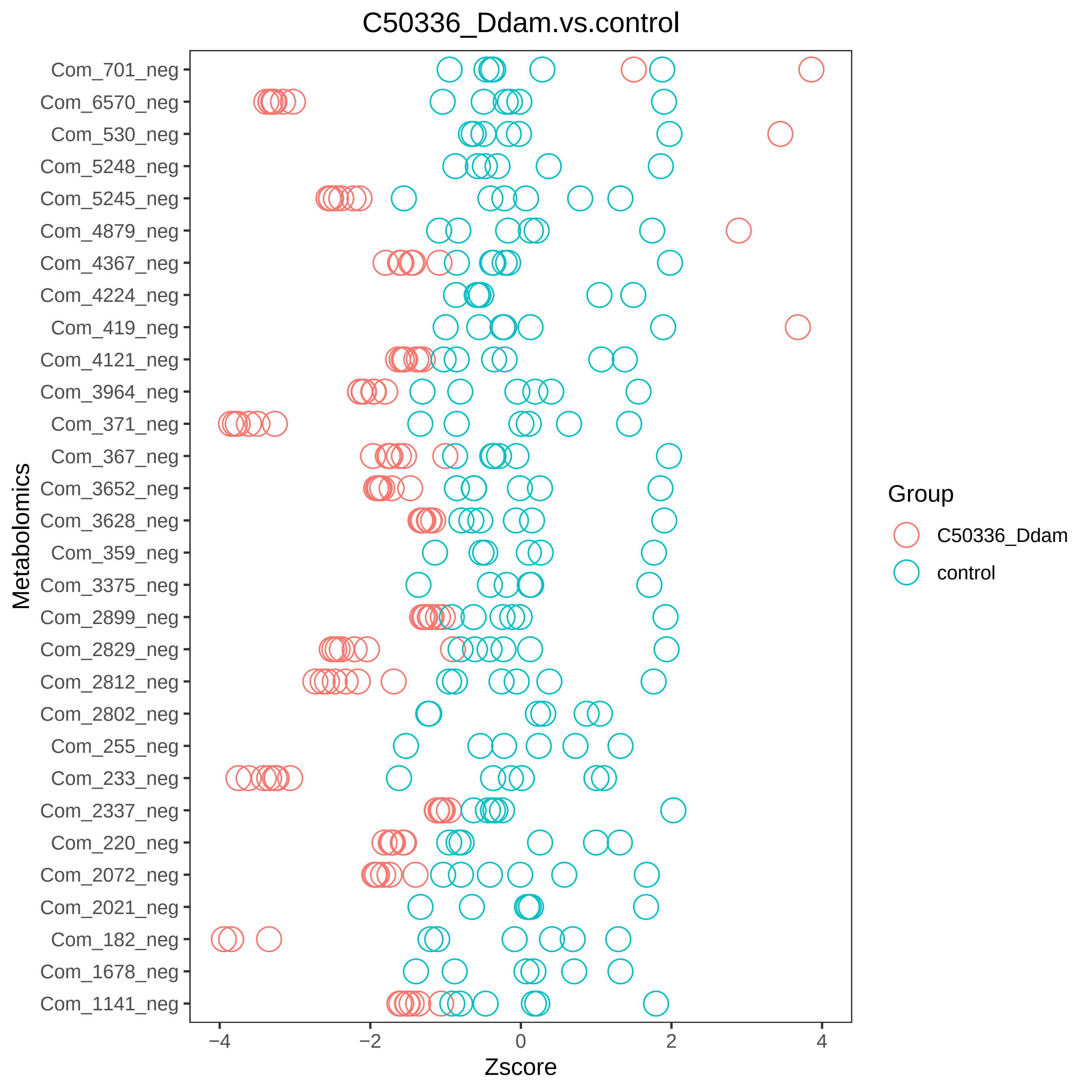

C50336_Ddam.vs.control
Com_701_neg
Com_6570_neg
Com_530_neg
Com_5248_neg
Com_5245_neg
Com_4879_neg
Com_4367_neg
Com_4224_neg
Com_419_neg
Com_4121_neg
Com_3964_neg
Com_371_neg
Com_367_neg
Com_3652_neg
Com_3628_neg
Com_359_neg
Com_3375_neg
Com_2899_neg
Com_2829_neg
Com_2812_neg
Com_2802_neg
Com_255_neg
Com_233_neg
Com_2337_neg
Com_220_neg
Com_2072_neg
Com_2021_neg
Com_182_neg
Com_1678_neg
Com_1141_neg
Metabolomics
−4
−2
0
2
4
Zscore
Group
C50336_Ddam
control
